# Prognostic factors of patients with intravascular large B cell lymphoma: a multicenter study in China

**DOI:** 10.3389/fonc.2025.1689028

**Published:** 2025-12-19

**Authors:** Yan Guo, Yi Zhong, Lixia Zhu, Fengping Zhou, Xuanru Lin, Xian Li, Xiufeng Wang, Yan Huang, Sun Wu, Guoqing Lv, Jinghang Zhang, Yi Zhao, Wenjun Wu, Xiujin Ye, Hanjin Yang, Jin Zhang, Kang Yu, Yun Liang, Zhen Cai, Jingsong He

**Affiliations:** 1 Department of Hematology, The First Affiliated Hospital of Xinxiang Medical University, Xinxiang, China; 2 Department of Hematology &Bone Marrow Transplantation Center, The First Affiliated Hospital, School of Medicine, Zhejiang University, Hangzhou, China; 3 Department of Hematology, Sir Run Run Shaw Hospital, Zhejiang University School of Medicine, Hangzhou, China; 4 Department of Hematology, The First Affiliated Hospital of Wenzhou Medical University, Wenzhou, China; 5 Department of Hematology, The Second Affiliated Hospital of Zhejiang University School of Medicine, Hangzhou, China; 6 Department of Pathology, The First Affiliated Hospital of Xinxiang Medical University, Xinxiang, China; 7 Department of Pathology, The First Affiliated Hospital, School of Medicine, Zhejiang University, Hangzhou, China

**Keywords:** intravascular large B-cell lymphoma, clinicopathological, treatment, poor prognostic, China

## Abstract

Intravascular large B-cell lymphoma (IVLBCL) is a rare and highly aggressive lymphoma, but current knowledge is still inadequate. We retrospectively analyzed 50 IVLBCL patients from five Chinese tertiary hospitals in China between 2017 and 2024. Hemophagocytic variant (HV) patients showed worse performance status, universal B symptoms, more bone marrow infiltration, higher mortality, pancytopenia, elevated inflammatory markers (CRP, LDH, ferritin), hypoglobulinemia and hypogammaglobulinemia. Among 46 treated patients, CR/CRu rate was 71% (27/38). The 2-year OS was 65.5%, significantly worse in HV vs. classical variant (CV) (43.3% vs. 76.4%, *P* = 0.007). Multivariate analysis identified CNS involvement (HR = 10.86, *P* < 0.001), HV subtype (HR = 1.91, *P* = 0.018), and nodal organs involvement (HR = 5.26, *P* = 0.052) as poor prognostic factors. IVLBCL exhibits marked heterogeneity, with HV and CNS involvement conferring dismal outcomes. This study provides key diagnostic/therapeutic insights for IVLBCL in China, warranting prospective trials to validate prognostic models and optimize therapies.

## Introduction

Intravascular large B-cell lymphoma (IVLBCL) is a rare subtype of diffuse large B-cell lymphoma with unique pathological features. It often presents with diagnostic errors or delays due to clinical heterogeneity (1). IVLBCL has three variants including cutaneous, classical variant (CV) and hemophagocytic variant (HV) (2–4). Retrospective studies showed that over 70% of patients are diagnosed at Ann Arbor stage IV and median survival approximately one year, indicating an extremely poor prognosis (1, 2, 5, 6). Therefore, enhancing the understanding of IVLBCL, establishing early diagnostic strategies, and optimizing treatment approaches are crucial for improving patient outcomes. This multicenter, retrospective study systematically analyzed IVLBCL cases diagnosed in several Chinese hematology centers to summarize the clinicopathological characteristics, treatment responses, and prognostic factors of IVLBCL, providing valuable insights for clinicians and contributing Chinese data to support the precision management of this rare disease.

## Methods

Patients with histologically confirmed IVLBCL according to WHO-HAEM5 criteria were retrospectively identified from five Chinese tertiary hospitals between 2017 and 2024. Collected information included: demographic characteristics, clinical symptoms, laboratory parameters, imaging techniques, results of bone marrow aspiration/biopsy, staging information, treatment regimens, and clinical outcomes. The Ann Arbor staging system was used in disease staging. The survival outcomes of patients were followed up to October 31, 2024. Progression-free survival (PFS) was defined as the time from diagnosis to disease progression, death, or the last follow-up. OS was defined as the time from diagnosis to death or the last follow-up.

This study was approved by the Ethical Review Committee of the First Affiliated Hospital of Zhejiang University School of Medicine (Approval No. ZDYY-LS-2024Y1351-K) and conducted in accordance with the principles of the Declaration of Helsinki. Patient identity information was kept anonymous prior to data analysis.

### Statistical analysis

Categorical variables are presented as frequencies (%), continuous variables as medians (ranges). Group comparisons used χ² or Fisher’s exact test in categorical variables and Mann-Whitney U-test in continuous variables. Continuous variables were dichotomized via clinical cutoff values or ROC curve analysis. Survival analysis employed Kaplan-Meier method with log-rank test and Cox regression analyses (univariate/multivariate). *P* < 0.05 was considered statistically significant; *P* < 0.1 in univariate analysis entered multivariate model. All analyses were performed using SPSS v.25 software.

## Results

### Clinical and laboratory characteristics

#### Clinical characteristics

This study analyzed 50 IVLBCL patients with median age 61 years (range: 32-82; 54% male), including 58% patients over 60 years old, and with median diagnosis delay of 75.5 days (range: 21-1132). Initial manifestations were diverse: B symptoms (36/50, 72%; predominantly fever), performance status decline (18%), neurological deficits (14%;including limb dyskinesia/paresthesia, dysautonomia, psychiatric abnormalities, hearing loss, and syncope), cutaneous rashes/nodules (30%), mass-effect symptoms (24%; including respiratory compromise and abdominal distension), and incidental detection (10%).

The cohort comprised two subtypes: classical variant (CV, 33/50, 66%) and hemophagocytic variant (HV, 17/50, 34%). HV group showed worse performance status (*P* = 0.009), universal B symptoms(*P* = 0.002), Bone marrow infiltration(*P*<0.001) and higher mortality (*P* = 0.011). No significant differences existed in sex, age distribution, staging, IPI, CNS involvement, Nodal organs or Nodal involvement between variants (**Table 1**).

**Table 1:** The Clinical and laboratory characteristics in two variants.

Characteristic	Total (n=50)	CV (n=33)	HV (n=17)	*P-value*
Demographics & staging				
Sex(Male)	27 (54.0%)	16 (48.5%)	11 (64.7%)	0.276
Age(>60 years)	29 (58.0%)	20 (60.6%)	9 (52.9%)	0.603
ECOG PS (2–4 scores)	**39 (78.0%)**	**22 (66.7%)**	**17 (100.0%)**	**0.009**
B symptoms	**36 (72.0)**	**19 (57.6%)**	**17 (100.0%)**	**0.002**
Ann Arbor Staging(III-IV)	45 (90.0%)	28 (84.8%)	17 (100.0%)	0.152
IPI(3–5 scores)	43 (86.0%)	26 (78.8%)	17 (100.0%)	0.080
CNS involvement	7 (14.0%)	6 (18.2%)	1 (5.9%)	0.398
Bone marrow infiltration	**33(66.0%)**	**16(48.5%)**	**17(100.0%)**	**<0.001**
Nodal Organs involvement	33(66.0%)	19(57.6%)	14(82.4%)	0.321
Nodal involvement	12(24.0%)	9(27.3%)	3(17.6%)	0.510
Laboratory parameters (median, range)				
WBC (x10^9^/L)	**5.14 (1.60-14.21)**	**5.90 (2.54-14.21)**	**4.06 (1.60-9.80)**	**0.027**
Neutrophils (x10^9^/L)	**3.43 (0.40-11.72)**	**3.62 (1.59-11.72)**	**2.47 (0.40-5.09)**	**0.014**
Lymphocytes (x10^9^/L)	0.90 (0.18-2.39)	0.91 (0.36-2.39)	0.80 (0.18-2.30)	0.068
Monocytes (x10^9^/L)	0.53 (0.03-2.87)	0.54 (0.03-1.66)	0.49 (0.15-2.87)	0.321
Hemoglobin (g/L)	**98.5 (53.0-153.0)**	**105.0 (53.0-153.0)**	**90.0 (62.0-149.0)**	**0.006**
Platelets (x10^9^/L)	**120.5 (30.0-392.0)**	**180.0 (43.0-392.0)**	**71.0 (30-146.0)**	**<0.001**
Albumin (g/L)	**30.4 (19.2-53.7)**	**33.6 (22.6-53.7)**	**26.6 (19.2-36.5)**	**0.002**
Globulin (g/L)	26.7 (16.0-48.8)	29.9 (16.0-39.6)	23.9 (18.9-48.8)	0.022
CRP (mg/L) (n=49)	**58.7 (0-174.5)**	**25.5 (0-156.1)**	**82.5 (37.7-174.5)**	**0.001**
ESR (mm/h) (n=32)	41.0 (2.0-140.0)	33.0 (2.0-140.0)	44.0 (6–118)	0.347
LDH (U/L)	**666** (120–4685)	**457** (120–4685)	**875 (304-3389)**	**0.014**
β2-MG (mg/L) (n=36)	3.91 (1.27-9.70)	4.0(1.27-9.70)	3.0 (3.80-5.32)	0.987
D-dimer (μg/L) (n=45)	970 (176-5980)	847 (176-5980)	1690 (294-5230)	0.056
Ferritin (μg/L) (n=43)	**943.7 (101.2-5332)**	**743.3 (101.2-743.3)**	**2321.8 (636.4-53321)**	**<0.001**
Immunoglobulins & cytokines				
IgG (mg/dL) (n=34)	**1185 (646-2523)**	**1290 (702-2523)**	**1054 (646-1487)**	**0.009**
IgA (mg/dL) (n=34)	240.0 (38.0-496.0)	230 (38-496)	277 (114-430)	0.484
IgM (mg/dL) (n=34)	64 (19-271)	65 (23-271)	54 (19-202)	0.348
IL-17A (pg/mL) (n=21)	1.50 (0.10-78.90)	0.99 (0.10-57.14)	3.07 (0.10-78.90)	0.189
IL-2, IL-4, IL-6, IL-10, TNF-α, IFN-γ(n=25)	No significant differences between groups.			All *P* > 0.05

Data are shown as the median (range) and n (%); Nodal Organs are defined as the lymph nodes and the spleen.

CV, Classic variant; HV, Hemophagocytic variant; ECOG PS, Eastern Cooperative Oncology Group performance status; IPI, International Prognostic Index; CNS, central nervous system; WBC, white blood cell; CRP, C-reactive protein; ESR, erythrocyte sedimentation rate; LDH, lactate dehydrogenase; β2-MG,β2-microglobulin; IL, interleukin; TNF-α, tumor necrosis factor-α; IFN-γ, interferon-γ; Ig, immunoglobulin. Emphasis: Key variables with significant differences (P < 0.05) between CV and HV groups are highlighted in bold. The HV column for the most critical differentiators (ECOG PS, B symptoms, Bone marrow infiltration, Platelets, CRP, Ferritin, IgG) is further emphasized with bold italics to draw visual attention to the pronounced abnormalities in the HV subtype.

#### Laboratory characteristics

Laboratory parameters revealed lower WBC (*P* = 0.027), neutrophils (*P* = 0.014), hemoglobin (*P* = 0.006), platelets (*P* < 0.001), and albumin (*P* = 0.002) in the HV group, alongside elevated CRP (*P* = 0.001) and LDH (*P* = 0.014). All HV cases had bone marrow infiltration and thrombocytopenia, compared to 48.5% (16/33) and 42.4% (14/33) in the CV group, respectively. These results highlight distinct clinical and laboratory profiles, underscoring the aggressive nature of HV (**Table 1**).

The study analyzed immunoinflammatory markers including D-dimer, ferritin, interleukins (IL-2, IL-4, IL-6, IL-10, IL-17A), tumor necrosis factor-α (TNF-α), interferon-γ (IFN-γ), and immunoglobulins (IgA, IgG, IgM). The HV group showed significantly higher ferritin (2321.8 vs 743.3 μg/L, *P* < 0.001) compared to CV group. CV patients had higher IgG levels (1290 vs 1054 mg/dL, *P* = 0.009). No other cytokines differed significantly between groups. These findings highlight distinct inflammatory profiles in IVLBCL subtypes (**Table 1**). EBV positivity was higher in HV (26.7%) than CV (11.1%), although this difference was not statistically significant (*P* = 0.204).

#### Pathological characteristics

**Figure 1:**
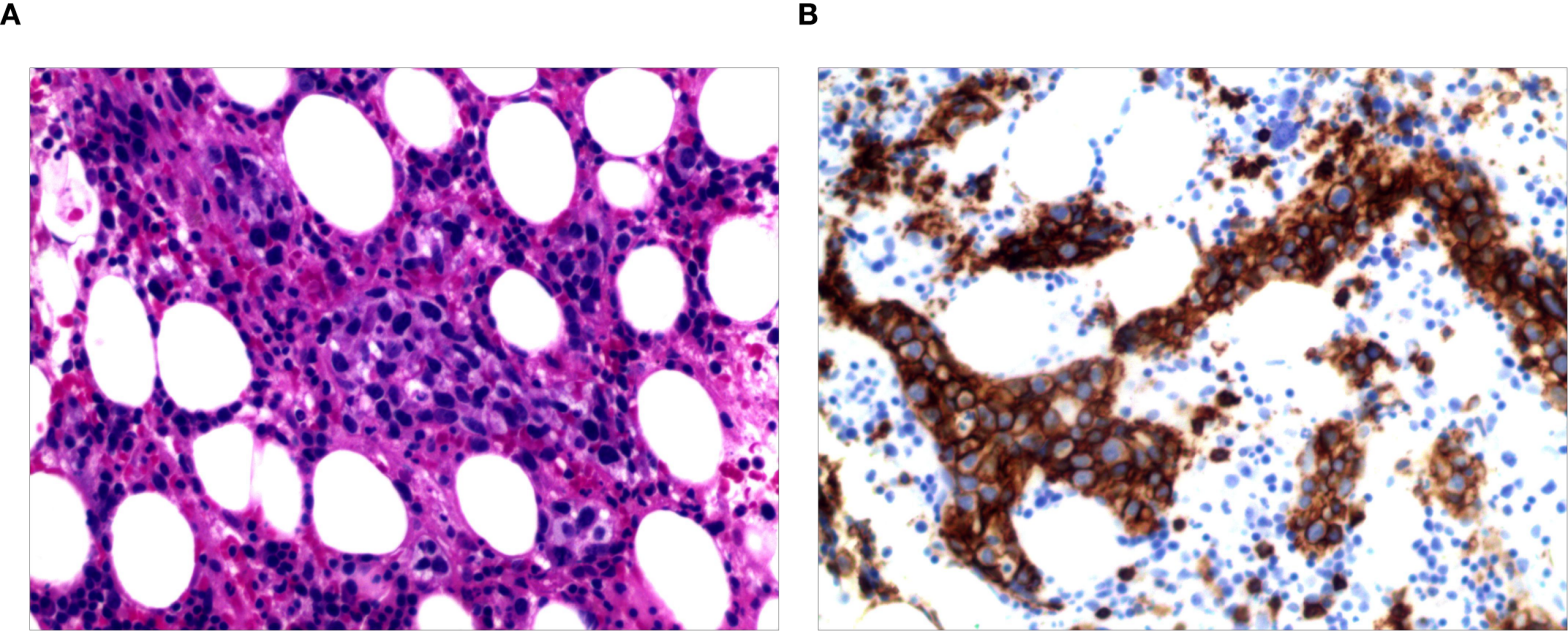
**(A)** H&E staining demonstrating vascular sinuses filled with lymphoma cells; **(B)** Immunohistochemical staining for CD20 is positive.

Biopsy sites included bone marrow (30%), skin (20%), lung (12%), CNS (10%), adrenal gland (8%), liver (6%), kidney (4%), uterus/uterus and vagina (4%), with single positive cases in muscle, prostate, and ileocecal region. Non-GCB subtype predominated (78.3%) with no difference between clinical variants. Tumor cells universally expressed CD20 (**Figures 1A, B**), with frequent positivity for CD19 (95.7%), CD79a (100%), MUM1 (83.8%), and BCL2 (92.1%), while CD10 (17.5%) and CD30 (10%) were less common. HV cases showed trends toward higher CD5 (53.8% vs 34.6%) and c-MYC (72.7% vs 60%) expression compared to CV, though statistically insignificant. Notably, CD5+ tumors strongly correlated with c-MYC expression (85.7% vs 35.7% in CD5−, *P* = 0.007) and trended toward BCL2 co-expression (100% vs 83.3%, *P* = 0.055). c-MYC/BCL2 dual expression occurred in 64.5% of cases. EBER and cyclinD1 were uniformly negative. These findings highlight IVLBCL’s aggressive B-cell phenotype, frequent dual-hit-like profiles, and potential CD5/c-MYC interplay, suggesting biological distinctions between variants despite overlapping morphology.

### Treatment an efficacy

Four untreated HV patients died early from disease progression (n=2), sudden death (n=1), and respiratory failure (n=1). Among 46 treated patients, 91.5% received rituximab-containing regimens and 93.5% received CHOP-like regimens. Additional agents included BTK inhibitors and/or lenalidomide (n=12), PD-1 inhibitors (n=3), and high-dose methotrexate (HD-MTX) (n=11, including 4/7 CNS cases). Of 38 evaluable patients, 71% achieved complete response/unconfirmed complete response (CR/CRu),18.4% partial response (PR), and 7.9% stable disease (SD). One CV patient achieved durable response after R-CHOP plus lenalidomide/HD-MTX followed by ASCT. Early mortality was exclusive to HV cases, highlighting their aggressive course. The overall response rate to CHOP-based therapy was 89.4%, with ASCT demonstrating potential for long-term response in selected cases.

A subgroup of 12 patients received therapy incorporating BTK inhibitors (BTKi) and/or lenalidomide (Len) during their course of treatment. The specific distribution was: BTKi alone (n=5), Len alone (n=2), and the combination of BTKi and Len (n=5). These novel agents were utilized across various settings, including salvage therapy for relapsed/refractory (R/R) disease and as part of frontline treatment for high-risk patients. In this subgroup, the overall response rate (ORR) was 83.3% (10/12), with a complete response (CR/CRu) rate of 66.7% (8/12). Treatment was generally well-tolerated.

Furthermore, 12 patients received maintenance therapy with these agents (Len, n=5; BTKi, n=5; BTKi/Len combination, n=1; rituximab, n=1) for a duration of 4 to 24 months following first-line treatment. Among 17 HV patients, 4 died early without treatment, and 13 received CHOP-like regimens (12 with rituximab, 2 with HD-MTX, 4 with BTKi).

CNS prophylaxis was administered to patients perceived to be at high risk for CNS relapse. Among the 46 treated patients, 11 (23.9%) received systemic high-dose methotrexate (HD-MTX) as CNS-directed prophylaxis. Of the 7 patients with established CNS involvement at diagnosis, 4 received HD-MTX as part of their therapeutic regimen. Intrathecal chemotherapy was not routinely administered for prophylaxis in this cohort.

### Survival analysis and prognostic factors

#### Survival outcomes

**Figure 2:**
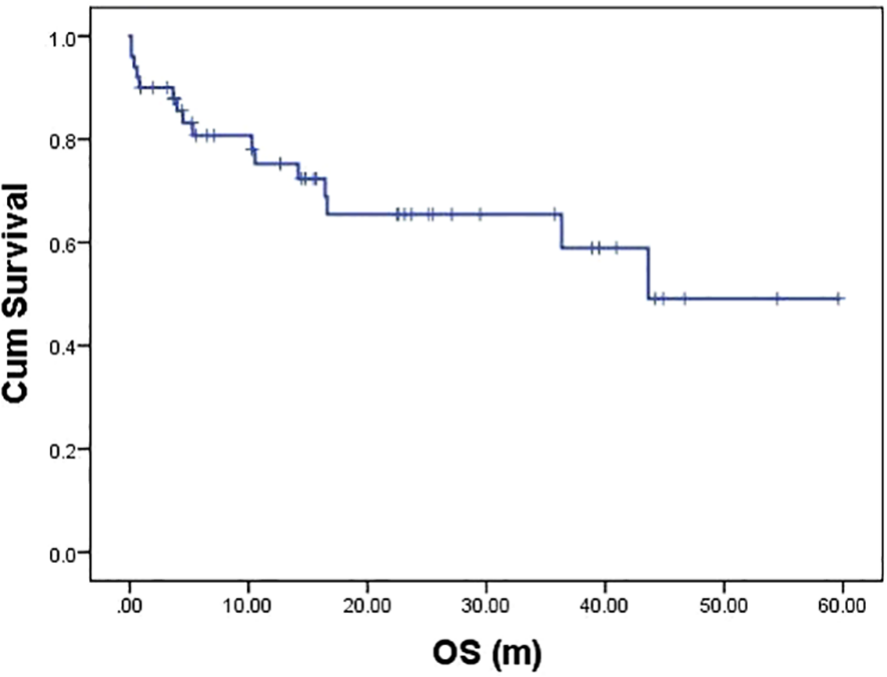
Overall survival of the entire IVLBCL cohort (n=50).

**Figure 3:**
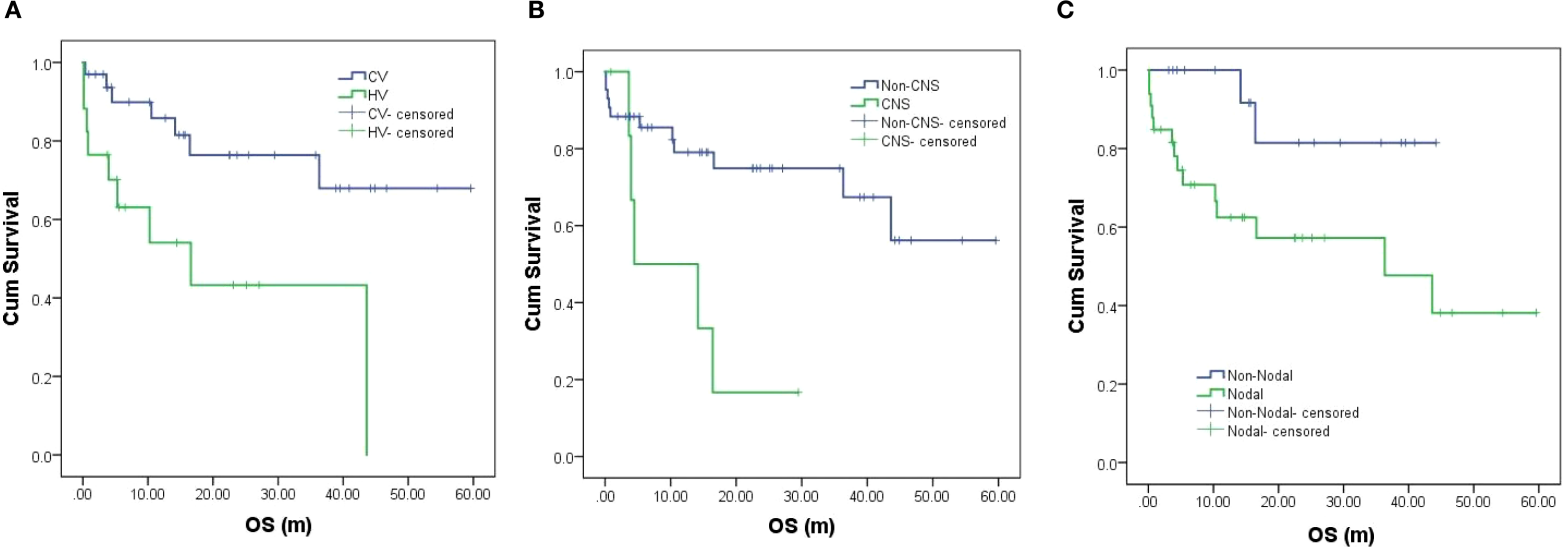
**(A–C)** Survival by **(A)** clinical subtype, **(B)** CNS involvement, and **(C)** nodal organs involvement.

**Figure 4:**
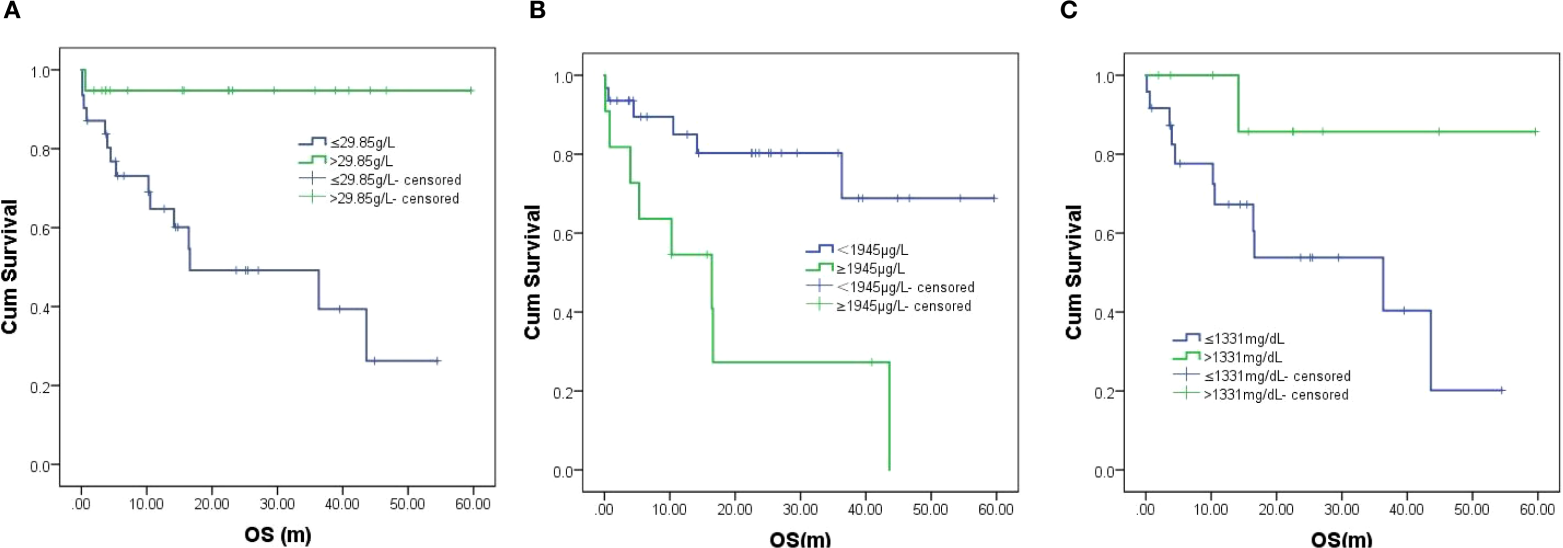
**(A–C)** Survival by **(A)** globulin level, **(B)** D-dimer level, and **(C)** IgG level. Left to right follow by **Figures 3A–C** in first row. Left to right follow by **(A–C)** in the second row.

With a median follow-up of 14.3 months (range: 0.13-59.60), 15 deaths occurred (13 lymphoma-related, 1 sudden cardiac death, and 1 severe infection). The 2-year OS was 65.5% (**Figure 2**), with significant differences between subtypes: CV patients showed superior outcomes (2-year OS 76.4%) versus HV (43.3%, *P* = 0.007) (**Figure 3A**, **Table 2**). CNS involvement was particularly detrimental (median OS 4.5 months vs NR, p=0.006), as was nodal organs involvement (median OS 36.3 months vs NR, *P* = 0.035) (**Figures 3B, C**, **Table 2**). Peripheral blood cytopenias (WBC ≤3.7×10^9^/L, neutrophils ≤2.6×10^9^/L, lymphocytes ≤1.11×10^9^/L) and low globulin (≤29.85 g/L) predicted inferior survival (all *P* < 0.05) (**Figure 4A**, **Table 2**). Maintenance therapy showed a trend toward improved outcomes (2-year OS 91.7% vs 54.7%, *P* = 0.076).Elevated D-dimer (≥1945 μg/L) correlated with worse survival (median OS 16.4 months vs NR, *P* = 0.002). Higher IgG (>1331 mg/dL) showed a protective trend (median OS NR vs 36.3 months, *P* = 0.058) (**Figures 4B, C**, **Table 2**).

**Table 2:** Univariate analysis of survival in IVLBCL patients (n=50).

Variables	Median OS (range)	2-year OS (%)	P
Clinical subtype			0.007
Classic variant	NR	76.4	
Hemophagocytic variant	16.6 (0.5~32.7)	43.3	
CNS involvement			0.006
No	NR	74.9	
Yes	4.5 (0-16.7)	16.7	
Nodal Organs involvement			0.035
No	NR	81.5	
Yes	36.3 (3.3-69.4)	57.2	
WBC (x10^9^/L)			0.045
≤3.7	16.4 (0.3-32.6)	44.1	
>3.7	NR	75.0	
Neutrophils (x10^9^/L)			0.008
≤2.6	14.2 (5.2-23.2)	40.0	
>2.6	NR	78.4	
Lymphocytes (x10^9^/L)			0.028
≤1.11	36.3 (12.2-60.5)	57.8	
>1.11	NR	88.9	
Monocytes (x10^9^/L)			0.067
≤0.38	16.6 (7.5-25.7)	40.9	
>0.38	NR	77.4	
Globulin (g/L)			0.004
≤29.85	16.6(0-38.6)	49.2	
>29.85	NR	94.7	
D-dimer (μg/L)			0.002
<1945	NR	82.1	
≥1945	16.43 (0.77-32.09)	27.3	
IgG (mg/dL)			
≤1331	36.33 (8.02-64.64)	53.8	0.058
>1331	NR	85.7	

OS, overall survival; NR, not reached.

**Figures 3** & **4**. Kaplan-Meier survival curves.

#### Multivariate analysis

CNS involvement (HR 10.86, *P* < 0.001), clinical variant (HR 1.91, *P* = 0.018), and nodal organs involvement (HR 5.26, *P* = 0.052) emerged as independent prognostic factors (**Table 3**).

**Table 3:** Cox regression analysis of risk factors affecting OS in IVBCL patients (n=50).

	Univariable cox		Multivariable cox	
	HR (95%CI)	*P*	HR (95%CI)	*P*
Nodal organs involvement	4.34 (0.98-19.19)	0.053	5.26 (0.98-28.12)	0.052
Clinical subtype	1.92 (1.16-3.17)	0.011	1.91 (1.12-3.27)	0.018
CNS involvement	4.18 (1.39-12.62)	0.011	10.86 (2.92-40.39)	<0.001
Neutrophils (x10^9^/L)	0.28 (0.10-0.77)	0.013		
Lymphocytes (x10^9^/L)	0.14 (0.02-1.08)	0.059		
Monocytes (x10^9^/L)	0.40 (0.14-1.11)	0.077		
Globulin (g/L)	0.09 (0.01-0.69)	0.020		

#### Key negative findings

No survival impact was observed for sex, age, ECOG PS, B symptoms, Ann Arbor stage, IPI score, EBV DNA status, or most laboratory parameters (hemoglobin, platelets, albumin, CRP, LDH).

This analysis identifies CNS involvement as the strongest negative predictor, followed by HV subtype and nodal organs involvement. The findings underscore the prognostic value of clinical subtype, disease distribution, and inflammatory markers in IVLBCL.

## Discussion

The HV Patient, prevalent in Asians including our cohort, features hemophagocytic syndrome, inflammatory responses, and frequent marrow infiltration (2, 4-7). In our cohort, all HV patients showed poor performance status (ECOG PS ≥2), advanced-stage disease (Ann Arbor III-IV), and high-risk IPI scores (≥3), with frequent marrow involvement. They exhibited characteristic cytopenias (neutropenia, lymphocytopenia, anemia, and thrombocytopenia) alongside elevated inflammatory markers (CRP, LDH, ferritin, D-dimer) and hypoalbuminemia, consistent with established HV features (2, 7). HV patients demonstrated significant hypoglobulinemia, particularly IgG reduction. Potential mechanisms include hepatic dysfunction, immune hyperactivation, or multi-organ failure. Unlike typical hemophagocytic syndrome, our HV cases showed no EBV association or survival impact.

Histopathology remains the gold standard for diagnosis of IVLBCL, though its variable presentation challenges biopsy site selection (1). Early multi-site biopsies, guided by PET/CT (though not exclusionary if negative), are essential for diagnosis (8, 9). For occult cases, marrow biopsies and random skin biopsy (RSB) are key when more than 5 clinical features present (unexplained fever/altered mental status/hypoxemia/cytopenia/LDH>800/IL-10 >95.65 pg/mL etc.) (10–14). Our data confirm marrow biopsy yield (30%) and IL-10’s diagnostic value (84%).

IVLBCL typically shows intravascular lymphoma cells (resembling DLBCL morphology) with occasional extravascular spread, expressing pan-B-cell markers, which key features include CD5+ (38%), MUM1+ (75-80%), and high Ki-67 index (4, 15). Our cohort confirmed these patterns, with HV showing non-significant trends for higher CD5+ and lower CD10+ rates than CV. CD5+ correlated with cMYC expression and showed poorer 2-year OS than CD5-, warranting further validation.

IVLBCL lacks standardized treatment, typically following DLBCL regimens. Rituximab-based therapy (e.g., R-CHOP) improves survival, though CNS relapse remains common (25% at 1–3 years) (6, 16–19). While PRIMEUR-IVL study reduced CNS relapse to 3% and validated in Chinese cohorts (14, 20). Our R-CHOP-based approach achieved 65.5% 2-year OS (vs historical 11.5-33%) (5–7). Molecular profiling reveals frequent NF-κB pathway mutations (MYD88/CD79B), supporting BTKi combinations (e.g., ZR-CHOP) currently under investigation (21–24). While ASCT consolidation shows promise (2-year PFS/OS 83%/89%), maintenance therapy (lenalidomide/BTKi) requires further study (25–27). In this study, only a CV patient achieved long-term progression-free survival after ASCT and 12 patients received maintenance therapy with lenalidomide and/or BTKi, which may have contributed to improved OS outcomes.

IVLBCL has a poor prognosis. Poor prognostic factors include age >60, ECOG PS ≥2, stage IV disease, thrombocytopenia (<150×10^9^/L), elevated LDH (≥700 U/L), CNS involvement, and hemophagocytic syndrome (5–7). In our cohort, clinical subtype significantly influenced outcomes: CV patients had superior OS, whereas CNS involvement with median survival of 4.5 months and nodal disease predicted worse survival. Additionally, Cytopenias often linked to hemophagocytic syndrome, including leukopenia, neutropenia, and lymphocytopenia were all associated with poorer OS. Hypoglobulinemia, particularly in HV patients, correlated with worse OS, likely due to infection-related mortality (28). Multivariate Cox regression analysis confirmed clinical subtype, CNS involvement, and nodal disease as independent adverse prognostic factors.

We hypothesize that nodal involvement in IVLBCL may be a marker of higher systemic tumor burden or a more disseminated disease state, even beyond the characteristic intravascular confinement. It could reflect biological aggressiveness, potentially associated with the phenotypic features observed (e.g., CD5 and c-MYC expression). Furthermore, nodal disease might facilitate access to richer lymphatic and vascular networks, promoting further dissemination. Its identification as an independent poor prognostic factor in our multivariate analysis underscores that IVLBCL with nodal spread represents a distinct, high-risk clinicopathological entity that warrants more intensive therapeutic strategies.

While the HV variant’s aggressive biology (CD5+, c-MYC+) might predispose to CNS spread (29), the observed lower incidence in our HV group could be influenced by several factors. The small sample size of the HV subgroup (n=17) limits the power to detect a significant difference. Furthermore, there might be a competing risk phenomenon; HV patients often present with rapidly progressive systemic illness, severe cytopenias, and multi-organ failure, leading to early death before CNS involvement becomes clinically apparent or can be thoroughly investigated. In contrast, CV patients might survive long enough for CNS disease to manifest or be detected.

Although limited by the small sample size and heterogeneous treatment lines, the promising efficacy observed in this cohort suggests that regimens containing BTKi and/or Len represent a viable and potent therapeutic strategy for IVLBCL. These findings strongly support further investigation in prospective clinical trials.

This multicenter Chinese study identified key clinical/pathological differences between HV/CV subtypes and prognostic impacts of nodal involvement. Diagnostic strategies emphasized repeated biopsies and cytokine profiling. Limitations included retrospective design, treatment heterogeneity, and potential inter-center bias. Future prospective studies should refine diagnostic/therapeutic approaches, establish prognostic models, and explore novel immunotherapies for IVLBCL.

## Data Availability

The raw data supporting the conclusions of this article will be made available by the authors, without undue reservation.

## References

[1] Ponzoni M Campo E Nakamura S . Intravascular large B-cell lymphoma: a chameleon with multiple faces and many masks. Blood. (2018) 132:1561–7. doi: 10.1182/blood-2017-04-737445, PMID: 30111607

[2] Murase T Nakamura S Kawauchi K Matsuzaki H Sakai C Inaba T . An Asian variant of intravascular large B-cell lymphoma: clinical, pathological and cytogenetic approaches to diffuse large B-cell lymphoma associated with haemophagocytic syndrome. Br J haematology. (2000) 111:826–34., PMID: 11122144

[3] Ferreri AJ Dognini GP Campo E Willemze R Seymour JF Bairey O . Variations in clinical presentation, frequency of hemophagocytosis and clinical behavior of intravascular lymphoma diagnosed in different geographical regions. Haematologica. (2007) 92:486–92. doi: 10.3324/haematol.10829, PMID: 17488659

[4] Alaggio R Amador C Anagnostopoulos I Attygalle AD Araujo IBO Berti E . The 5th edition of the world health organization classification of haematolymphoid tumours: lymphoid neoplasms. Leukemia. (2022) 36:1720–48. doi: 10.1038/s41375-022-01620-2, PMID: 35732829 PMC9214472

[5] Seegobin K Li Z Alhaj Moustafa M Majeed U Wang J Jiang L . Clinical characteristics, prognostic indicators, and survival outcomes in intravascular lymphoma: Mayo Clinic experience (2003-2018). Am J hematology. (2022) 97:1150–8. doi: 10.1002/ajh.26635, PMID: 35713565 PMC9541514

[6] Liu Z Zhang Y Zhu Y Zhang W . Prognosis of intravascular large B cell lymphoma (IVLBCL): analysis of 182 patients from global case series. Cancer Manage Res. (2020) 12:10531–40. doi: 10.2147/CMAR.S267825, PMID: 33122951 PMC7591067

[7] Ferreri AJ Campo E Seymour JF Willemze R Ilariucci F Ambrosetti A . Intravascular lymphoma: clinical presentation, natural history, management and prognostic factors in a series of 38 cases, with special emphasis on the ‘cutaneous variant’. Br J haematology. (2004) 127:173–83. doi: 10.1111/j.1365-2141.2004.05177.x, PMID: 15461623

[8] Schönau V Vogel K Englbrecht M Wacker J Schmidt D Manger B . The value of (18)F-FDG-PET/CT in identifying the cause of fever of unknown origin (FUO) and inflammation of unknown origin (IUO): data from a prospective study. Ann rheumatic diseases. (2018) 77:70–7. doi: 10.1136/annrheumdis-2017-211687, PMID: 28928271

[9] Matsukura K Hokkoku K Shiraoka A Yang L Takahashi Y Hatanaka Y . Increased uptake on (18) F-fluorodeoxyglucose positron emission tomography/computed tomography is indicative of occult skin lesions in a patient with intravascular large B-cell lymphoma. J Dermatol. (2018) 45:e254–e5. doi: 10.1111/1346-8138.14302, PMID: 29569289

[10] Enzan N Kitadate A Tanaka A Matsue K . Incisional random skin biopsy, not punch biopsy, is an appropriate method for diagnosis of intravascular large B-cell lymphoma: a clinicopathological study of 25 patients. Br J Dermatol. (2019) 181:200–1. doi: 10.1111/bjd.17603, PMID: 30609011

[11] MacGillivary ML Purdy KS . Recommendations for an approach to random skin biopsy in the diagnosis of intravascular B-cell lymphoma. J cutaneous Med surgery. (2023) 27:44–50. doi: 10.1177/12034754221130257, PMID: 36205174 PMC9902969

[12] Matsue K Abe Y Kitadate A Miura D Narita K Kobayashi H . Sensitivity and specificity of incisional random skin biopsy for diagnosis of intravascular large B-cell lymphoma. Blood. (2019) 133:1257–9. doi: 10.1182/blood-2018-11-887570, PMID: 30647028 PMC6450056

[13] Matsue K Abe Y Narita K Kobayashi H Kitadate A Takeuchi M . Diagnosis of intravascular large B cell lymphoma: novel insights into clinicopathological features from 42 patients at a single institution over 20 years. Br J haematology. (2019) 187:328–36. doi: 10.1111/bjh.16081, PMID: 31267524 PMC6900202

[14] Zhang Y Wang L Sun J Wang W Wei C Zhou D . Serum interleukin-10 as a valuable biomarker for early diagnosis and therapeutic monitoring in intravascular large B-cell lymphoma. Clin Trans Med. (2020) 10:e131. doi: 10.1002/ctm2.131, PMID: 32634257 PMC7418806

[15] Turner JJ Morton LM Linet MS Clarke CA Kadin ME Vajdic CM . InterLymph hierarchical classification of lymphoid neoplasms for epidemiologic research based on the WHO classification (2008): update and future directions. Blood. (2010) 116:e90–8. doi: 10.1182/blood-2010-06-289561, PMID: 20699439 PMC2993636

[16] Shimada K Matsue K Yamamoto K Murase T Ichikawa N Okamoto M . Retrospective analysis of intravascular large B-cell lymphoma treated with rituximab-containing chemotherapy as reported by the IVL study group in Japan. J Clin Oncol Off J Am Soc Clin Oncol. (2008) 26:3189–95. doi: 10.1200/JCO.2007.15.4278, PMID: 18506023

[17] Ferreri AJ Dognini GP Bairey O Szomor A Montalbán C Horvath B . The addition of rituximab to anthracycline-based chemotherapy significantly improves outcome in ‘Western’ patients with intravascular large B-cell lymphoma. Br J haematology. (2008) 143:253–7. doi: 10.1111/j.1365-2141.2008.07338.x, PMID: 18699850

[18] Takahashi H Nishimaki H Nakanishi Y Hamada T Nakagawa M Iizuka K . Clinical impact of central nervous system-directed therapies on intravascular large B-cell lymphoma: A single institution’s experience. EJHaem. (2022) 3:467–70. doi: 10.1002/jha2.380, PMID: 35846032 PMC9176124

[19] Shimada K Murase T Matsue K Okamoto M Ichikawa N Tsukamoto N . Central nervous system involvement in intravascular large B-cell lymphoma: a retrospective analysis of 109 patients. Cancer science. (2010) 101:1480–6. doi: 10.1111/j.1349-7006.2010.01555.x, PMID: 20412122 PMC11158344

[20] Shimada K Yamaguchi M Kuwatsuka Y Matsue K Sato K Kusumoto S . Rituximab, cyclophosphamide, doxorubicin, vincristine, and prednisolone combined with high-dose methotrexate plus intrathecal chemotherapy for newly diagnosed intravascular large B-cell lymphoma (PRIMEUR-IVL): long-term results of a multicentre, single-arm, phase 2 trial. EClinicalMedicine. (2025) 80:103078. doi: 10.1016/j.eclinm.2025.103078, PMID: 39968389 PMC11833343

[21] Chen C Di Y Zhuang Z Cai H Jia C Wang W . Plasma circulating tumour DNA is a better source for diagnosis and mutational analysis of IVLBCL than tissue DNA. J Cell Mol Med. (2024) 28:e18576. doi: 10.1111/jcmm.18576, PMID: 39054569 PMC11272604

[22] Gonzalez-Farre B Ramis-Zaldivar JE Castrejón de Anta N Rivas-Delgado A Nadeu F Salmeron-Villalobos J . Intravascular large B-cell lymphoma genomic profile is characterized by alterations in genes regulating NF-κB and immune checkpoints. Am J Surg pathology. (2023) 47:202–11. doi: 10.1097/PAS.0000000000001978., PMID: 36221796 PMC9833110

[23] Shimada K Yoshida K Suzuki Y Iriyama C Inoue Y Sanada M . Frequent genetic alterations in immune checkpoint-related genes in intravascular large B-cell lymphoma. Blood. (2021) 137:1491–502. doi: 10.1182/blood.2020007245, PMID: 33512416 PMC7976508

[24] Zhang Y Jia C Wang W Zhang L Cao X Li J . The interim analysis from a prospective single-center phase 2 study of zanubrutinib plus R-CHOP in treat-naïve intravascular large B cell lymphoma. Blood. (2021) 138:3563–. doi: 10.1182/blood-2021-152417

[25] Kato K Mori T Kim SW Sawa M Sakai T Hashimoto H . Outcome of patients receiving consolidative autologous peripheral blood stem cell transplantation in the frontline treatment of intravascular large B-cell lymphoma: Adult Lymphoma Working Group of the Japan Society for Hematopoietic Cell Transplantation. Bone marrow transplantation. (2019) 54:1515–7. doi: 10.1038/s41409-019-0491-7, PMID: 30809035

[26] Meissner J Finel H Dietrich S Boumendil A Kanfer E Laboure G . Autologous hematopoietic stem cell transplantation for intravascular large B-cell lymphoma: the European Society for Blood and Marrow Transplantation experience. Bone marrow transplantation. (2017) 52:650–2. doi: 10.1038/bmt.2016.339, PMID: 27991887

[27] Nowakowski GS Chiappella A Gascoyne RD Scott DW Zhang Q Jurczak W . ROBUST: A phase III study of lenalidomide plus R-CHOP versus placebo plus R-CHOP in previously untreated patients with ABC-type diffuse large B-cell lymphoma. J Clin Oncol Off J Am Soc Clin Oncol. (2021) 39:1317–28. doi: 10.1200/JCO.20.01366, PMID: 33621109 PMC8078325

[28] Brazel D Grant C Cabal A Chen WP Pinter-Brown L . Baseline immunoglobulin G and immune function in non-Hodgkin lymphoma: a retrospective analysis. Front Immunol. (2024) 15:1334899. doi: 10.3389/fimmu.2024.1334899, PMID: 38745669 PMC11091275

[29] Epperla N Zayac AS Landsburg DJ Bock AM Nowakowski GS Ayers EC . High-grade B-cell lymphoma, not otherwise specified: CNS involvement and outcomes in a multi-institutional series. Blood Adv. (2024) 8:5355–64. doi: 10.1182/bloodadvances.2024013791, PMID: 39189932 PMC11568788

